# Psychiatric role in physician-assisted death requests – a study protocol for a literature review

**DOI:** 10.1192/j.eurpsy.2023.2201

**Published:** 2023-07-19

**Authors:** R. Barranha, A. R. Ferreira, E. Gonçalves, L. Fernandes

**Affiliations:** 1 Psychiatry Service, Centro Hospitalar Universitário de São João; 2 Department of Clinical Neurosciences and Mental Health; 3 CINTESIS@RISE, Faculty of Medicine, University of Porto; 4 Palliative Care Service, Centro Hospitalar Universitário de São João; 5 Department of Community Medicine, Information and Health Decision Sciences (MEDCIDS), Faculty of Medicine, University of Porto, Porto, Portugal

## Abstract

**Introduction:**

The prospect of a medium-term approval of physician-assisted death in Portugal raises relevant ethical and deontological issues that need to be addressed, namely the framework of psychiatric assessments in this process. Such assessments are undermined by the lack of scientific precision in the methods used to determine decision-making capacity, making it possible for the final decision to be affected by psychiatrists’ personal beliefs. As such, outlining scientific evidence and legislation pieces defining the psychiatrists’ role and scope is of utmost importance to frame this debate.

**Objectives:**

To synthetize the accumulated evidence worldwide regarding the psychiatrists’ involvement in the global process of physician-assisted death requests by reviewing scientific literature, published protocols, official reports and international promulgated or amended legislation related to hasten death practices.

**Methods:**

*PubMed*, *Scopus*, *Web of Science*, *PsycInfo* and *Google Scholar* electronic bibliographic databases will be searched for eligible articles, as well as grey literature, using the following search terms: Psychiatry AND (Euthanasia OR (Suicide AND Assisted)). Official governments’ and countries health authorities’ websites will also be searched for relevant reports and legislation documents, as well as right-to-die organizations and akin associations. No language, date of publication, or geographical restrictions will be applied. The full text of potentially relevant results will be retrieved from the different sources for review after screening titles and abstracts. This two-stage process will be conducted independently by two researchers. Outcomes of interest will be the descriptions of psychiatric role in the process of physician-assisted death requests, assessment methods, and measurement techniques used.

**Results:**

Given the fact that physician-assisted death is legalized only in a few jurisdictions, we believe the number of eligible results will be limited. Data will be extracted and a descriptive summary of the evidence will be provided. We anticipate finding a significant variability, but also to identify points of consensus. The findings will be published in a peer-review indexed journal and presented at national and international conferences.

**Conclusions:**

To our knowledge, this is the first review of both, scientific published literature, and international legislation on the role of psychiatrists in physician-assisted death requests. We hope to provide an international overview to frame the public debate by pinpointing the most consensual assessment methodology, allowing to design an optimized assessment protocol before the implementation of the law in Portugal.

**Disclosure of Interest:**

None Declared

